# Cavitation During Superplastic Forming

**DOI:** 10.3390/ma4071271

**Published:** 2011-07-08

**Authors:** John Campbell

**Affiliations:** University of Birmingham, Birmingham, B15 2TT, UK; E-Mail: jc@campbelltech.co.uk; Tel.: +44 (0) 1531 636 077.

**Keywords:** superplasticity, cavitation, casting defect, bifilm

## Abstract

Cavitation is the opening of pores during superplastic forming, typically at grain boundary triple points or on second phase grain boundary particles during slip of grain boundaries. Theories for the initiation of cavitation are reviewed. It seems that cavitation is unlikely to occur by processes *intrinsic* to metals such as dislocation mechanisms or point defect condensation. It is proposed that cavitation can only occur at non-bonded interfaces such as those introduced *extrinsically* (*i.e.*, from the *outside*) during the original casting of the metal. These defects, known as oxide bifilms, are naturally introduced during pouring of the liquid metal, and are frozen into the solid, often pushed by dendritic growth into grain boundaries where they are difficult to detect because of their extreme thinness, often measured in nanometres. Their unbonded central interface acts as a crack and can initiate cavitation. Second phase precipitates probably do not nucleate and grow on grain boundaries but grow on bifilms in the boundaries, explaining the apparent association between boundaries, second phase particles and failure initiation. Improved melting and casting techniques can provide metal with reduced or zero bifilm population for which cavitation would not be possible, promising significant improvements in superplastic behaviour.

## 1. Introduction

Superplastic forming is usually limited by the development of voids, a process commonly known as cavitation. Voids are seen to form at grain boundaries, often at grain corners or triple points, or at second phase particles in the boundaries during the process of grain boundary slip [1]. The growth and linking of voids leads to progressive failure. Intrinsic to these suggested nucleation sites is the assumption that the grain boundaries are weak and/or sufficient stress can be developed at the grain corners or second phases to nucleate cavities. The assumptions underlying these failure mechanisms are questioned in this paper.

## 2. An Alternative Theory for Cavitation

An alternative explanation for the formation of cavitation is explored in this review. It is based on the assumption that grain boundaries are strong as a result of their relatively perfect atomic contact between grains (a small percentage of loss because of different degrees of perfection of contact can be neglected, since strengths will in any case be expected to be in the tens of gigapascals, similar to the ultimate strength of relatively perfect metal grains). Cavitation is therefore not expected occur at grain boundaries.

Cavitation would be expected at a grain boundary if parts of the boundary were somehow unbonded. Such unbonded areas are to be expected as a result of the presence of bifilms. Bifilms are doubled-over oxide films entrained into the melt during the casting of the metal. The entrainment process necessarily brings together the dry surfaces of any surface oxide on the liquid, resulting in an unbonded interface that acts as a crack in the liquid (all submerged oxide films will be necessarily be double in this way; careful thought will confirm to the reader that it is not possible to entrain a single oxide film). The outer surfaces of the double film are of course perfectly wetted in the sense of being in perfect atomic contact with the melt, having grown atom by atom from the liquid when the oxide was originally growing on the melt surface. These outer surfaces appear to be excellent substrates for the nucleation and growth of second phases and intermetallics [2,3].

Because of the turbulent handling methods used in foundries and cast houses, oxide bifilms are predicted to be widespread in Al alloys, and can exist as densely packed populations.

Bifilms can also result from the powder metallurgy consolidation process, in which individual grains of powder, each exhibiting an oxide film, are pressed together. Somewhat similar processes are to be expected in spray forming. In this paper the relatively specialized powder and spray routes are not further considered; since the greater tonnage of superplastic alloys are produced by casting, this route is dealt with in detail.

Thus it is proposed that superplastic material should continue to flow plastically without the occurrence of cavitation, allowing reduction of area of the material to neck down 100%. The fact that this does not occur arises because of the initiation of cavitation at bifilms in grain boundaries, or bifilms associated with second phase particles. Without bifilms, as unbonded interfaces acting as pre-existing cracks, boundaries and second phase particles would not exhibit cavitation since the metallic bond is so strong. The evidence for these predictions is considered below.

There have been a number of elegant, classical theoretical studies to compute the theoretical strength of liquid and solid metals. In the short review of this topic by the author [4] the treatment by Fisher [5] stands out with its powerful logic. From energy considerations, using macroscopic concepts such as surface tension, he finds the critical radius at which a pore is stable. Since pores will grow one atom at a time by statistical fluctuations, pores smaller than the critical radius will tend to disappear. Only exceptionally will a long chain of favourable energy fluctuations produce a pore exceeding the critical radius. When this rare event happens, estimated by rate theory, the pore has the potential to grow to observable size. His formula for the tensile strength of liquid gives values of 3.1 GPa for liquid Al and 7.0 GPa for liquid Fe. The theoretical strength of solid metals is expected to be even higher than those of their liquid phases because the interatomic distances are slightly closer. Clearly, both liquid and solid metals are expected, with good reason, to have high strengths.

Whereas many texts now conclude that some pre-existing pore must now be postulated, such as a pocket of gas trapped in a recess in an inclusion, such assumptions pre-suppose the very problem we are attempting to explain. How could a void or gas pocket occur in a solid produced by solidification? The atomic movements during the reorganisation of the liquid metal into a solid are only small fractions of an atomic diameter; the structure of the liquid is that of a randomly close packed solid, and the structure of the solid is, of course, regular, but otherwise very similar, with quite similar interatomic spacings. The high forces that keep the atoms together effectively forbid the opening of a void.

These extremely high stresses for the ‘homogeneous’ nucleation of pores or cracks might, of course, be reduced in the presence of a poorly-wetted substrate that would allow ‘heterogeneous’ nucleation. (It is probably worth pointing out that the solid/liquid interface is of course well wetted, being in perfect atomic contact with both liquid and solid phases, and so not a favoured substrate for the creation of volume defects). However, for conditions of the worst possible wetting, assuming the highest contact angles ever recorded, in the region of 160 degrees, the nucleation stress is predicted to be reduced by a factor of nearly 20. Thus the fracture stresses for both liquid and solid metals is somewhat reduced, but remains high [4].

Significantly, the stresses remain in the range 10^3^ to 10^4^ times higher than can be met during solidification, since, as every foundry person knows, a poorly fed Al alloy casting can collapse forming external sinks under only atmospheric pressure (0.1 MPa), indicating the limit to which internal tensile stress can be supported. Thus the tensile stresses sufficient to create volume defects in castings cannot be generated, simply because inter-atomic forces are too high to allow pores to open and hot cast metal near its melting point is in general too weak to support such stresses.

Even in some solid metals at room temperature there has been direct evidence for over 40 years that cracks and pores cannot be formed homogeneously [6]. Transmission electron microscope observations of the condensation of a supersaturation of vacancies in a lattice might be expected to form cavities in the same way that condensation of supersaturated solutes can form second phases. However, for all metals studied so far, this is not true. TEM observations of quenched fcc metals, including Al, Ag and Au, indicates that condensation of vacancies does occur, but instead of the formation of vacancy discs or three-dimensional voids, the lattice collapses under its own interatomic forces, consolidating to create dislocation rings or stacking fault tetrahedra depending on the stacking fault energy.

These historical demonstrations in the classical face centred cubic lattices have been more recently extended to other more complex metals. In more recent electron radiation studies of vacancy condensation in Fe [7], Mo [8], Zr [9] and U [10] voids were never reported; only dislocation loops were observed. Recent Molecular Dynamics (MD) simulations confirm this behaviour [11,12,13] showing how clusters of up to 45 vacancies collapse unstably to stacking fault tetrahedra.

Only when extremely high isostatic tensions are applied to a metal does it seem possible for the metal to cavitate. MD studies by Milstein [13] indicate that a tensile stress of over 15GPa is required to stabilize a void in Ni, causing it to grow explosively to promote failure. Void growth studies by Meyers *et al.* [14] used reflected shock waves in Cu to find voids formed at grain boundaries only when the tensile stress exceeded 37 GPa.

Clearly, we can conclude that volume defects in metals is not possible either as a result of solidification or at high temperatures during the application of normal tensile stresses during superplastic forming. The inter-atomic forces can only be overcome to create voids at huge stresses corresponding to the theoretical rupture stress of the metal. The absence of failure initiation mechanisms necessarily implies that tensile tests should result in either in very high tensile strengths corresponding to the theoretical strength of the solid, or extensive plastic flow, necking down to 100 % reduction in area.

## 3. Practical Defect Generating Mechanisms

It follows that conditions for failure are clear and logical: in general, failure can initiate in a metal only from interfaces that are unbonded (since atomic bonds are too strong to be broken). Since unbonded surfaces cannot be formed by intrinsic processes such as solidification or vapour phase deposition, or by plastic working of the solid (as discussed below) such interfaces have to be introduced from *outside* the metal. These are necessarily *extrinsic* features.

There are three main extrinsic defects: pores, bifilms and extrinsic (exogenous) inclusions [2]. All are effectively introduced in to the matrix by the impingement of surfaces during consolidation. Thus in power metallurgy routes such defects are necessarily formed. In casting the impingement of liquid surfaces during turbulence is unfortunately common, but not necessary, as is discussed further below.

**Figure 1:**
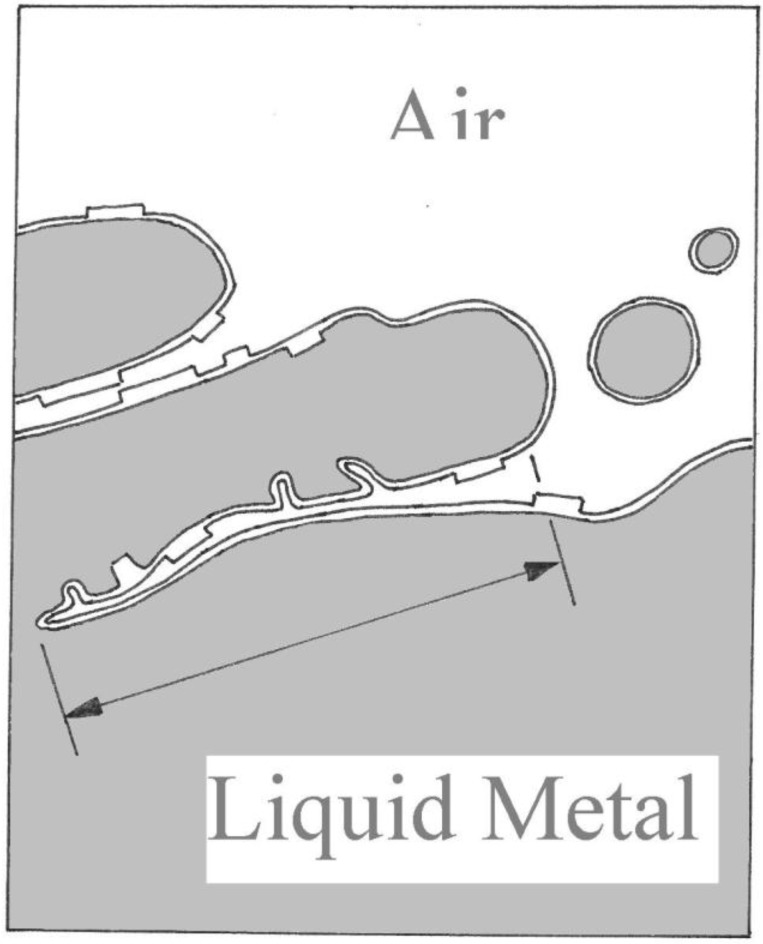
Creation of bifilms by surface turbulence.

**Figure 2:**
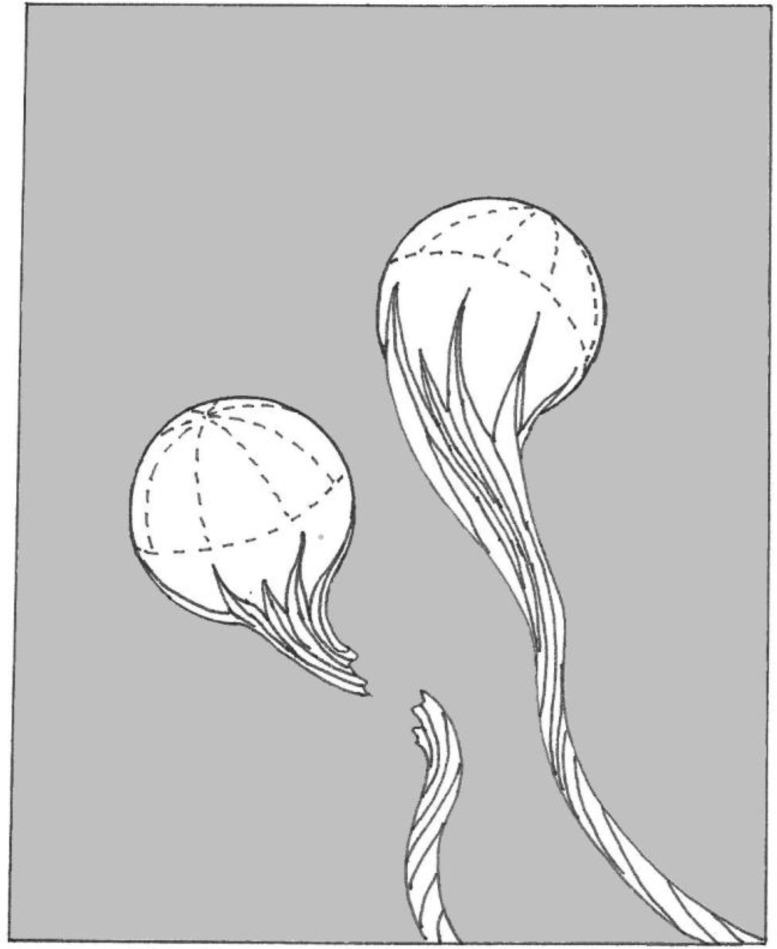
Schematic illustration of air bubbles rising in a liquid metal, their oxide sloughing off to create a bubble trail, a kind of long tubular bifilm.

**Figure 3:**
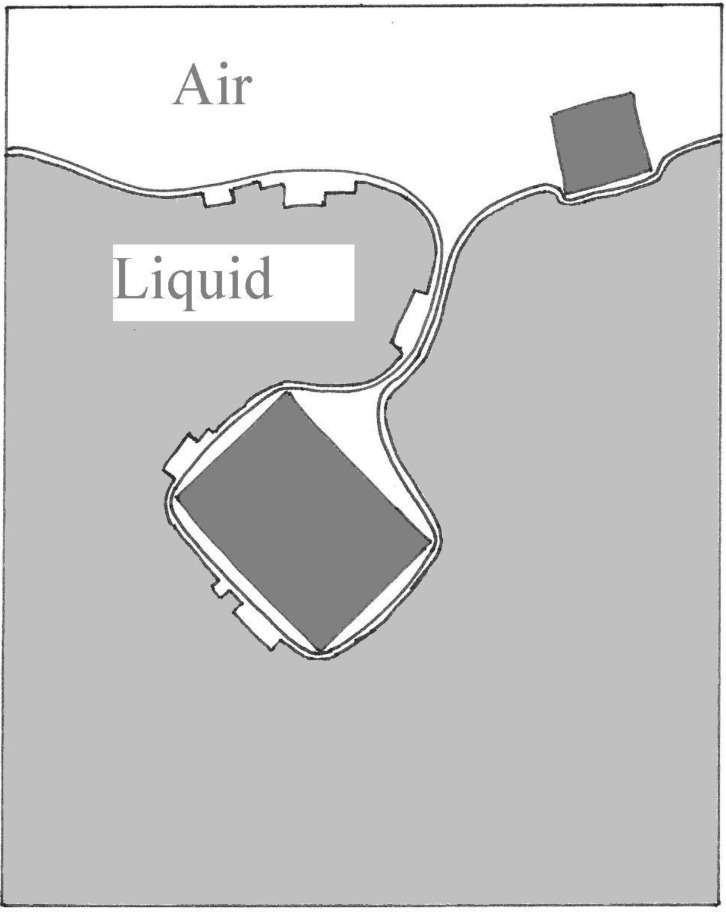
The entrainment of the surface oxide as an extrinsic inclusion penetrates the surface of a melt.

During casting the entrainment process incorporates bubbles, bifilms and extrinsic inclusions as the result of impinging droplets, or the folding over of breaking waves. The entrainment actions and the nature of the defects are illustrated in Figure 1, Figure 2 and Figure 3. Such defects, especially the bifilms, are introduced into the melt at every stir and every pour event. Also, a succession of such handling traumas adds its contribution to the total population of suspended defects in the liquid. Although the near-neutral density of the alumina bifilms in liquid aluminium ensures that these defects have a long life in suspension in the melt, severe bifilm problems can also be experienced in a wide variety of cast metals, including cast irons, stainless steels and Ni-based superalloys even when cast in so-called vacuum. These defects are, of course, subsequently frozen in to the solid.

The main difference between bubbles and bifilms is the amount of air they contain. Whereas bubbles are of course straightforwardly understood, their common association with a bubble trail (as a tubular bifilm) remaining in the wake of the bubble during its travels through the melt is not generally realised [2].

Despite its extensive unbonded interface (diameters are typically in the range micrometers to centimeters) the bifilm is generally overlooked because it is often so thin (usually in the range of nanometers to micrometers) as to be invisible to casual observation. The bifilm is, of course, usually an oxide, but can on occasions be a film of carbon, or nitride, *etc.* Its folded or collided origin necessarily results in its structure characterised by a double film with (i) unbonded inner faces, entrapping traces of residual air, and (ii) perfectly wetted exterior faces (originally the underside of the surface oxide film). All entrained oxide films necessarily have this double structure. These features explain their pivotal roles affecting the mechanical and metallurgical properties of castings and their wrought products.

The third variety of entrained defect, the extrinsic inclusion, has to enter the melt through the surface oxide, necessarily carrying with it a wrapping of the surface film, and thus remains isolated from the melt by the oxide and its entrapped layer of air. In effect it enjoys no bond with the matrix, and contrasts therefore with the *in-situ* intrinsic inclusion that has grown atom-by-atom from the melt, and thus remains in perfect atomic contact at all points. This phenomenon applies to all melt additions, including the oxide skins on the charge materials (that subsequently become double films when submerged) and alloy additions to the melt.

Studies to date have indicated that the population of bifilms in Al alloys [15] appears to be high, estimated to be often in the range 10^6^ to 10^9^ m^−3^. Steels [16,17] and Ni-base alloys [18] are expected to have similar populations. This at first sight may appear surprising in view that these features are not generally reported. The realization that such a dense population of defects is the norm in metals makes a re-interpretation of much accepted metallurgy highly desirable. Some recent instances include the widely different phenomena: (i) the fatigue of ductile irons initiated from magnesium silicate bifilms [19]; (ii) incipient melting and cracking in weld heat affected zones of Ni-base alloys [20]; (iii) corrosion of Al sacrificial anodes in sea water [21]; and (iv) porosity and viscosity of MMCs [22].

The bifilms in cast metals appear to survive considerable plastic working. Thus these casting defects influence the behaviour of many wrought products. Al alloys retain their unbonded regions even after the severe extrusion required to produce window frames, as is evident from the filiform corrosion, clearly seen by eye on unprotected extrusions, in which tens or hundreds of corrosion sites per square centimetre follow the elongated unbonded bifilms that tunnel through the metal, happening to intersect the surface from time to time to create a corrosion site. The survival of the bifilms during plastic working is probably the result of the reservoirs of air that remain trapped in the rucks and folds between the oxide surfaces. Thus any extension of the area of the interfaces by working is accompanied by simultaneous oxidation and nitridation of the freshly-created surfaces, preventing bonding until all the air is consumed. It is proposed that these unbonded surfaces are probably common in most wrought alloys, and constitute the defects necessary for failure by cavitation, brittle cracking, ductile tearing, or corrosion.

From much work carried out on the application of pressure during solidification, and from compaction during such processes as hot isostatic pressing of cast metals, the benefits to properties are almost certainly the result of the closing of defects such as pores and bifilms. There is evidence in many metals that pores and bifilms are easily and quickly closed, but are reluctant to bond or weld [23,24] which is to be expected from the great stability of some of their surface oxides and nitrides (exceptions to this include those oxides that react at hip temperatures [25]). Thus although the defects will remain weak in tension between their surfaces, the increased strength properties almost certainly arise from the fact that the defects are now closed; their contacting surfaces can now at least resist shear as a result of friction and jogs.

## 4. The Role of ‘Brittle’ Intermetallics and Second Phases

**Figure 4:**
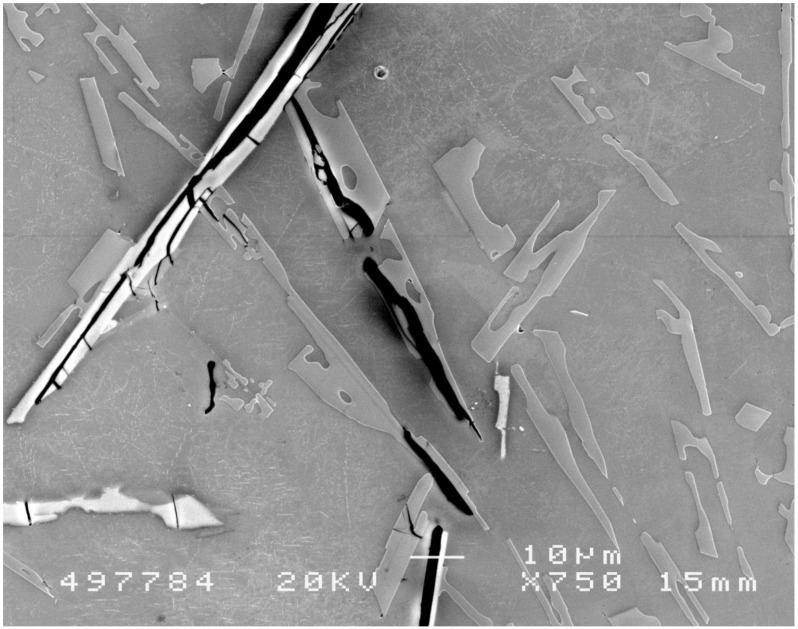
Beta-Fe particle (diagonally upper left) and Si particles (centre) in an Al-Si alloy casting, showing central and transverse cracks, and apparent decoherence from the matrix, consistent with their formation on oxide bifilms, and opening as a result of poor feeding conditions generating a hydrostatic tensile stress (Courtesy X. Cao).

Every primary intermetallic and primary second phase so far investigated appears to have formed on the wetted outer surfaces of a bifilm [2,3]. An image of beta-Fe particles and Si particles in Al-Si alloys is shown in Figure 4. The central cracks denote the location of the originating bifilm (the short transverse cracks on one side of the main crack are rucks and folds in one of the components of the main bifilm, since when folding together in a turbulent event, one component will always have a larger area than the other, and will therefore be forced to adopt additional wrinkles and creases, whereas its adjoining film will be mainly flat). The bifilms are expected to be present in every beta-Fe and silicon particle in the alloy, but are not always obvious. In Figure 4 the bifilms have been opened, appearing as cracks, because of inflation, possibly by some shrinkage or gas, during solidification. It seems likely that in the absence of such favoured substrates neither beta-Fe nor primary Si flakes will precipitate as primary phases but will be forced to appear at lower temperatures as constituents of eutectic phases. This is the proposed mechanism of modification of Si by Na and Sr, both of which are proposed to deactivate bifilms as substrates [26,27,28].

In general, it seems likely therefore that the appearance of cracked intermetallics is not an indication of brittleness. Intermetallics are known to be strong, and the forces involved during solidification of metals are generally a factor of 10^5^ to 10^6^ too small to cause fracture. The cracks merely denote the presence of an unbonded interface that is an integral feature of their favoured substrate. These considerations are corroborated by measured fracture strengths of Si particles in Al-Si alloys that have been shown to be as low as 200 MPa [29] compared to expected theoretical strengths of at least 30GPa [30,31,32,33].

It follows that classical physical (intrinsic) metallurgy would predict that an Al-Si eutectic alloy undergoing a tensile test would not exhibit a failure of a single Si particle. The particles would cluster together as the Al matrix plastically flowed until they impinged. At that point the Si particles themselves might start to deform plastically until the whole specimen finally parted by necking down to 100 % reduction in area. These counter-intuitive predictions will be astonishing to witness.

## 5. Decohesion

There are various experimental observations that support the view that failure under tensile stress does not initiate from those phases that have formed by precipitation from solution in the melt or matrix. The good bonding of the interface will preclude any possibility of separation. In contrast, those phases associated with an unbonded interface dragged into the melt from the liquid surface are likely to have almost zero contact with the surrounding matrix, and thus easily decohere, initiating a pore or crack.

Decohesion is also possible for those phases that have precipitated from solution, but which happen to have formed on only one side of a bifilm. The other side, now consisting of only the flimsy unbonded film is easily separated from the other half of the bifilm that is now firmly attached to the precipitate. Thus it appears that the precipitate is capable of nucleating a pore or crack.

The ability of a surface to resist decoherence from a matrix in perfect molecular contact was nicely demonstrated as long ago as 1867 by Gernez [34]. He showed that crystalline solids which had been grown in the liquid, and which had never been allowed to come into contact with air, were incapable of inducing effervescence in a liquid supersaturated with gas. Otherwise identical solids which had been allowed to dry always caused effervescence. In this experiment the decohering forces were, of course, relatively weak, but the principle seems sound. By analogy, but operating at higher strengths, the contact between intermetallics and the matrix from which they were formed will be atomically perfect, and thus will be strong.

The famous observations on NaCl crystals [35], brittle when crushed in air, but deforming in a ductile mode when compressed under water because surface flaws are dissolved away, is analogous to the condition of an intermetallic that had been formed *in-situ* in a melt, having an atomically smooth interface with the liquid that enjoys essentially perfect atomic contact, and so enjoying extreme resistance to decohering or fracturing.

Emamy and Campbell [36] compared the bonding between second phases and the matrix in two commercial metal matrix composites (MMCs) by solidifying a casting under the modest hydrostatic tension induced by the lack of feeding of a cylindrical shape. It was clear that the MMC formed by introducing SiC particles through the liquid surface, even when this was conducted under high vacuum to reduce oxide problems, exhibited significant decoherence from the matrix, creating a dispersion of fine pores. In contrast the MMC containing TiB_2_ particles which had been formed by *in-situ* reaction in the matrix were resistant to decoherence from the matrix despite being subjected to even higher hydrostatic tension (the opening of pores by decoherence of a large proportion of SiC particles to some extent relieved the tension experienced by the surviving SiC/matrix interfaces).

Bifilms, with their central unbonded interface, are pushed by dendrites, and therefore often finally reside in grain boundaries. Thus those boundaries containing bifilms will easily decohere (being effectively pre-cracked) during creep or superplastic flow. From observations of decohesion of such boundaries it is usually concluded that boundaries in general are weak, even though most boundaries do not decohere. Clearly, boundaries that do not contain bifilms will be expected to be strong; it may be appropriate to remind ourselves that virtually all of our major engineering alloys are full of grain boundaries: it is not conceivable that such features are weak.

## 6. Experimental Observations of Failure

A direct observation of bifilms in the Al-Mg alloy AA5083 appears to have been made by Kulas and coworkers [37] who studied the opening of voids occurring during grain boundary sliding. SEM observations revealed longitudinal filaments extending across the void and aligned in the direction of deformation. Unfortunately these authors did not analyse the filaments to confirm that they were in fact oxides, although it is difficult to imagine what other material could have been present to account for such observations.

Other authors working on the same alloy observed that grain boundary sliding during hot deformation produced pores adjacent to every Al_6_(Mn,Fe) particle over 10μm and larger, and adjacent to 60 per cent of MgSi particles in the size range 2–3 μm. Although once again the authors did not suspect that presence of oxides, if we assume that the intermetallics only precipitate on the wetted exterior faces of bifilms (which is behaviour that appears common to the precipitation of all intermetallics studied so far [2,3]) then the opening of the bifilm will provide the observed voids. (Conversely, voids would not be expected at an intermetallic/matrix interface in the absence of a bifilm because of the expected high tensile strength of this interface.)

In similar studies by microtomography on a super-austenitic stainless steel [38] every sigma phase particle was associated with at least one void.

Direct observations of the tensile deformation to rupture of single crystals of Fe-3%Si steel [33] revealed that plastic cavities were observed to be invariably associated with inclusions, particularly if these were located in shear zones. The cavities would be expected to form from the opening up of prior cracks associated with the presence of the bifilm on which the inclusions had formed. For material free from inclusions (implying an absence of bifilms) plastic cavities were not observed to open up at the intersection of slip zones or at any stage of plastic deformation. Failure occurred by massive sliding that occurred along one predominant plane. In such conditions of intense dislocation activity, one might expect failure by such lattice defect mechanisms as the dislocation pile-up. Clearly, this was not observed, and suggests support for the hypothesis that cracks cannot occur by intrinsic mechanisms such as a dislocation pile-up. In the case of a pile-up, any threatened opening of a cavity or a crack would be predicted to be thwarted by the triggering of additional dislocation activity, bringing in additional matrix material by the flow of dislocations from alternative directions.

Wu and Sandstrom [39] working on nucleation and growth of creep cavities in a 12Cr-Mo-V steel found that pores apparently initiated after only a few per cent of the total creep life, so early that it was highly suggestive that the pores pre-existed, but were initially invisible. Furthermore, at final rupture, the creep specimen was observed to contain cracks and pores throughout its volume, the pores often arranged into ‘strings’, suggestive of bifilms decorated with pores as might be expected from the irregular folding of the oxide, trapping air in patches across its area, as is indicated in Figure 1.

**Figure 5:**
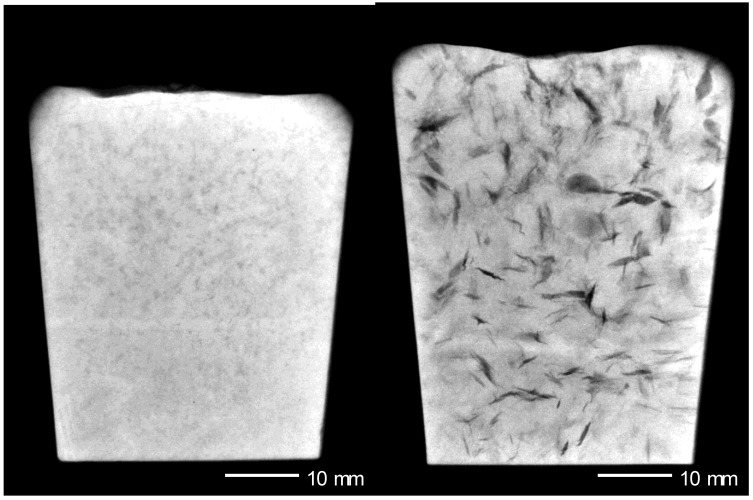
A radiographic image of a divided sample of the same A356 Al alloy poured into two 35 mm diameter stainless steel cups [41]. On the left the sample solidified under atmospheric pressure displays faint images of compact bifilms. On the right the bifilms have been opened up and straightened by subjecting the sample to a pressure of a tenth of an atmosphere, expanding the residual air between the opposite halves of the bifilm. Note the size and density of bifilms in this sample (Courtesy Simon Fox).

The extent of bifilm problems in cast metals is probably illustrated well by the experiments of Yu and Sun [40] in which vacuum melted Fe_3_Al intermetallic alloy was cast into sand moulds. The sand mould would have out-gassed prolifically, ensuring that the vacuum during pouring was extremely poor and oxidising. In addition, the slow rate of solidification in the sand moulds would have allowed time for the bifilms to unravel from their original fairly innocuous compact state after the maelstrom of pouring, unfolding to become serious engineering cracks [2,41]. (A similar unfolding of bifilms, transforming them from relatively harmless features into serious cracks is encouraged by reducing the pressure during freezing, thus expanding the residual air in the bifilms, as is clearly seen in Figure 5.) During subsequent attempts to forge the alloy the ingots were subject to severe cracking, sometimes falling into pieces under the forge. This work contrasts with that by Frommeyer *et al.* [42] who cast the identical alloy, but almost certainly into metal moulds, thus preserving the good vacuum in the vacuum chamber, and the rapid rate of freezing would preserve the compact convoluted form of the bifilms, greatly reducing their effectiveness as cracks. They extruded and swaged their ingots without difficulty, achieving a superplastic behaviour of 350 % elongation despite the large grain size and expectations of brittle behaviour.

## 7. Experimental Observations of Avoidance of Failure

**Figure 6:**
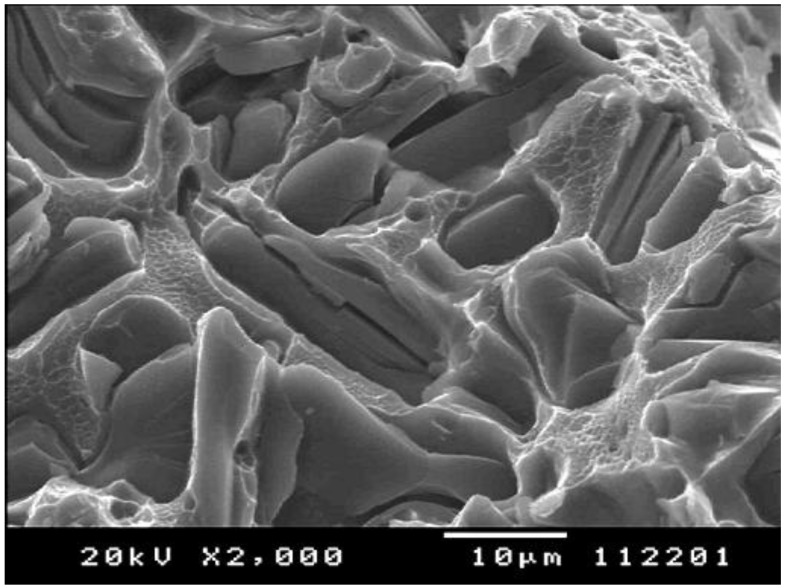
SEM image of a fracture surface of an Al-Si eutectic phase (Courtesy M. Tiryakioglu).

The theoretical prediction of 100 % reduction of area of metals free from extrinsic defects is supported on a microscale, as is evident in SEM images of ductile failure (Figure 6). The knife edged cusps at some locations between the cups and cones are regions that typify the failure of metals without defects. The failure mode exhibits necking to zero in this tiny cusp area. This microscale example appears similarly true on a macroscale in ‘cup and cone’ failure in tensile test pieces, where ductile necking to failure is only interrupted by the nucleation and growth of a central cracking region. Without the nucleation of the central crack, the test piece would continue to extend until 100 per cent reduction in area.

Accepting for a moment the high density of defects commonly present to initiate failure, if, despite their presence, conditions are applied to prevent the defects opening and propagating, then high tensile performance would be expected as though the defects were absent. For instance, when tensile tests are carried out under pressures approaching the ultimate failure strength of the material, elongation continues to 100 % reduction of area. Bridgeman [43] showed that as the hydrostatic pressure on a steel specimen approached 2.67 GPa the RA rose to 100 per cent. This would be expected if cavities were prevented from opening, thus artificially simulating conditions for a metal without initiating sites.

In conclusion it needs to be stated that modern melting technology is now capable of producing bifilm-free melts in most metals and alloys. Similarly, such very clean melts can be now cast without turbulence that would re-introduce a second population of bifilms. These practices are beginning to be adopted, and substantial achievements in terms of improved cast products are beginning to be reported [44].

## Conclusions

It is postulated that extrinsically-sourced defects entrained during manufacture dominate the failure of metals, and that these features constitute the main, if not the only, source of failure initiation in both cast and wrought metal. Thus:

1. Powder metallurgy processes necessarily result in extrinsic permanent damage.
2. Unnecessarily poor melting and casting techniques cause casting defects creating extrinsic permanent damage in metals.
3. Solidification does not lead to the creation of extrinsic damaging defects (although may subsequently enhance the damaging effects of casting defects, for instance, by expanding pores or cracks by hydrostatic tension arising from solidification shrinkage, or by the segregation of gases).
4. Porosity or cracks have an *extrinsic* origin in the casting process and can lead to failure of metals.
5. Probably most if not all types of failure only occur from entrained unbonded interfaces, mainly bifilms. (Failures that appear to have initiated by decoherence or fracture of extrinsic inclusions will in fact have originated on its associated bifilm acquired during entrainment, and failures associated with *in-situ* precipitated inclusions originate from the bifilm forming their preferred substrate for formation and growth.)
6. Extrinsic defects introduced during the manufacture of metals appear to survive significant plastic working processes, so that casting defects tend to be permanent in wrought metals.
7. In an appropriately cast and solidified metal, within the scope of current technology, a Griffith crack could not be formed and could not subsequently be generated by, for instance, plastic flow, and thus in general would not exist.

Thus metals cast without such defects would be expected to exhibit the following microstructural behaviour:

1. Intermetallics should never crack because although they may have little ductility, they are in general extremely strong (furthermore, most would never form as primary or secondary phases in the absence of a favourable entrained bifilm substrate).
2. Grain boundaries should never decohere in creep or superplastic forming.
3. Inclusions formed *in-situ* in the melt or matrix should never decohere from the matrix.
4. Failure of metals should therefore occur by brittle fracture only at the extremely high theoretical failure stress, or by ductile failure at extremely high elongations and 100% reduction of area.

In summary; many, if not most, metal failures, including cavitation during superplastic forming, originate from *extrinsic* defects, particularly bifilms entrained during casting or powder processing. Unbonded interfaces need not be entrained during the casting of metals so that premature failure leading to cavitation need not occur. Thus the potential for improved performance from both cast and wrought metals by improved melting and casting techniques is huge. The technology for improved metals and alloys relatively free from bifilms appears now to be understood, and is beginning to be implemented [16,17,44].

## References

[1] Perevezentsev V.N., Rybin V.V., Chuvil’deev V.N. (1992). The theory of structural superplasticity—IV. Cavitation during superplastic deformation. Acta Metall. Mater..

[2] Campbell J. (2006). Entrainment defects. Mater. Sci. Technol..

[3] Campbell J. (2004). Castings: The New Metallurgy of Cast Metals.

[4] Campbell J. (1968). The Solidification of Metals.

[5] Fisher J.C. (1948). The fracture of liquids. J. Appl. Phys..

[6] Kuhlmann-Wilsdorf D., Moser P., Corbel C., Lucasson P., Hautojarvi P. (1987). Lattice defects in quenched metals. Materials Science Forum.

[7] Sen Gupta A., Moser P., Bourret A., Corbel C., Naidu S.V., Sen P., Hautojarvi P. (1987). Vacancy defects in 3.0MeV electron irradiated Mo single crystal at 20K. Materials Science Forum.

[8] Hellio C., de Novion C.H., Marraud A., Boulanger L. (1987). Point defect clustering in electron and ion irradiated Zr alloys. Materials Science Forum.

[9] Weiberg C., Quere Y. (1987). Dislocation loops in irradiated uranium. Materials Science Forum.

[10] Uberuaga B.P., Hoagland R.G., Valone S.M., Voter A.F. (2007). Direct transformation of vacancy voids to stacking fault tetrahedra. Phys. Rev. Lett..

[11] Sabochick M.J., Yip S., Lan N.Q. (1987). Calculation of the properties of vacancy clusters in copper using a new, efficient energy minimization scheme. Mater. Sci. Forum.

[12] Traiviratana S., Bringa G.M., Benson D.J., Meyers M.A. (2008). Void growth in metals: Atomistic calculations. Acta Mater..

[13] Milstein F., Zhao J., Maroudas D. (2004). Atomic pattern formation at the onset of stress-induced elastic instability: Fracture *versus* phase change. Phys. Rev. B.

[14] Meyers M.A., Traiviratana S., Lubarda V.A., Benson D.J., Bringa E.M. (2009). The Role of Dislocations in the growth of nanosized voids in ductile failure of metals. JOM.

[15] Dispinar D., Akhtar S., Nordmark A., Di Sabatino M., Arnberg L. (2010). Degassing, hydrogen and porosity phenomena in A356. Mater. Sci. Eng. A.

[16] Puhakka R., Tiryakioglu M., Campbell J., Crepeau P.N. Premium quality super duplex stainless steel casting without secondary refining. TMS Annual Conference, 4th Shape Casting Symposium.

[17] Puhakka R., Tiryakioglu M., Campbell J., Crepeau P.N. Advanced methoding concepts for the gravity casting of steel alloys. TMS Annual Conference, 4th Shape Casting Symposium.

[18] Campbell J., Tiryakioglu M., Campbell J., Crepeau P.N. Review of defect behavior in Ni-based superalloys. TMS Annual Conference, 4th Shape Casting Symposium.

[19] Campbell J. (2008). Fatigue Fracture of Ductile Cast Iron. Int. J. Metalcast..

[20] Campbell J. (2009). Letter regarding HAZ cracking and properties of TRIP steels. Mater. Sci. Technol..

[21] Sina H., Emamy M., Saremi M., Keyvani A., Mahta M., Campbell J. (2006). The influence of Ti and Zr on electrochemical properties of aluminum sacrificial anodes. Mater. Sci. Eng. A.

[22] Emamy M., Abbasi R., Kaboli S., Campbell J. (2009). Fluidity of Al based metal matrix composites containing Al2O3 and SiC particles. Int. J. Cast Met. Res..

[23] Staley J.T., Tiryakioglu M., Campbell J., Crepeau P.N., Tiryakioglu M., Campbell J. (2007). Effect of Various HIP Conditions on Bifilms and Mechanical Properties in Aluminum Castings. Shape Casting: 2nd International Symposium.

[24] Griffiths W.D., Raiszadeh R., Omotunde A.O., Crepeau P.N., Tiryakioglu M., Campbell J. (2007). The effect of holding time on double oxide film defects in aluminium alloy castings. Shape Casting: 2nd International Symposium.

[25] Lumley R.N., Sercombe T.B., Schaffer G.B. (1999). Surface oxide and the role of magnesium during the sintering of aluminium. Metall. Mater. Trans. A.

[26] Campbell J. (2009). Discussion of “Effect of Strontium and Phosphorus on Eutectic Al-Si Nucleation and Formation of β -Al5FeSi in Hypoeutectic Al-Si Foundry Alloys”. Metall. Mater. Trans. A.

[27] Campbell J., Tiryakioglu M. (2010). Review of effect of P and Sr on modification and porosity development in Al-Si alloys. Mater. Sci. Technol..

[28] Tiryakioglu M., Campbell J., Staley J.T. (2003). The influence of structural integrity on the tensile deformation of cast Al–7wt.%Si–0.6wt.%Mg alloys. Scripta Mater..

[29] Griffiths J., Oliver E.C., Fitzpatrick M.E., Finlayson T.R., Viano D., Wang Q., Crepeau P.N., Tiryakioglu M., Campbell J. (2007). Stresses in the Eutectic Si Particles of Sr-Modified A356 Castings Loaded in Tension. Shape Casting: 2nd International Symposium.

[30] Orowan E. (1949). Fracture and strength of solids. Rep. Prog. Phys..

[31] Kelly A. (1966). Strong Solids.

[32] Gall K., Horstemeyer M.F., van Schilfgaarde M., Baskes M.I. (2000). Atomistic simulations on the tensile debonding of an aluminum–silicon interface. J. Mech. Phys. Solids.

[33] Govila R.K., Hull D. (1968). Necking and fracture of [110] 3%SiFe crystals at 293 and 473K. Acta Mater..

[34] Gernez M. (1867). On the nucleation of bubbles in liquid metals. Philos. Mag..

[35] Mendelson S. (1962). Role of surfaces in plastic flow of NaCl single crystals. II. J. Appl. Phys..

[36] Emamy M., Campbell J. (1995). Solidification shrinkage in MMCs. Cast Metals.

[37] Kulas M.A., Green W.P., Taleff E.M., Krajewski P.E., McNelley T.R. (2006). Failure mechanisms in superplastic AA5083 materials. Metall. Mater. Trans. A.

[38] Fonda R.W., Lauridsen E.M., Ludwig W., Tafforeau P., Spanos G. (2007). Two-Dimensional and Three-Dimensional Analyses of Sigma Precipitates and Porosity in a Superaustenitic Stainless Steel. Metall. Mater. Trans. A.

[39] Wu R., Sandstrom R. (1995). Creep cavity nucleation and growth in 12Cr-Mo-V steel. Mater. Sci. Technol..

[40] Yu X.Q., Sun Y.S. (2004). Hot working of Fe3Al based alloy. Mater. Sci. Technol..

[41] Frommeyer G., Derder C., Jimenez J.A. (2002). Superplasticity of Fe_3_Al(Cr). Mater. Sci. Technol..

[42] Fox S., Campbell J. (2000). Visualisation of oxide film defects during solidification of aluminium. Scr. Mater..

[43] Bridgeman P.W. (1952). Studies in Large Plastic Flow and Fracture (with Special Emphasis on the Effects of Hydrostatic Pressure.

[44] Campbell J. (2004). Casting Practice: The 10 Rules for Casting.

